# Comparative study of efficacy and safety: Biosimilar rituximab versus originator rituximab in the treatment of pemphigus

**DOI:** 10.1111/1346-8138.17329

**Published:** 2024-06-14

**Authors:** Sang Heon Shin, Jae Yeon Kim, Soo‐Chan Kim, Jong Hoon Kim

**Affiliations:** ^1^ Department of Medicine Yonsei University College of Medicine Seoul Korea; ^2^ Department of Dermatology, Gangnam Severance Hospital, Cutaneous Biology Research Institute Yonsei University College of Medicine Seoul Korea; ^3^ Department of Dermatology, Yongin Severance Hospital, Cutaneous Biology Research Institute Yonsei University College of Medicine Seoul Gyeonggi‐do Korea

**Keywords:** biosimilar, originator, pemphigus foliaceus, pemphigus vulgaris, rituximab

## Abstract

Rituximab is a monoclonal antibody that targets CD20 antigen in B cells. For pemphigus, rituximab has been highly effective in steroid‐sparing therapy for moderate to severe cases. Originator rituximab has demonstrated favorable treatment effects in patients with pemphigus, but its high cost remains a challenge. Biosimilar rituximab is expected to offer a potential solution. However, it is required for the comparative study of efficacy and safety between biosimilar and originator because all biosimilars may not be identical to the originator. In this study, we compared the treatment effects and safety of biosimilar (Truxima) and originator (MabThera) rituximab in patients with pemphigus. A final cohort of 52 patients in the MabThera group and 72 patients in the Truxima group was enrolled. Except for the intravenous immunoglobulin administration rate, there were no differences in baseline characteristics between the two groups, and for the purpose of comparing efficacy, investigations into time to complete remission, total steroid intake to complete remission, and total steroid intake for 6 months following rituximab treatment revealed no significant differences between the two groups. Truxima can be considered a relatively affordable alternative treatment option for pemphigus, offering cost‐effectiveness to patients who are indicated for the treatment with MabThera.

## 1 INTRODUCTION

Pemphigus, a potentially lethal autoimmune disease, is characterized by bullae and erosion formation due to acantholysis.^1^ Its pathophysiology is driven by autoantibodies against desmogleins, cell surface proteins on keratinocytes. Pemphigus can be classified into representative subtypes, pemphigus vulgaris (PV) and pemphigus foliaceus (PF), based on the desmoglein isotypes targeted by autoantibodies.^2^

Rituximab, a monoclonal antibody targeting CD20 antigen on B cells, originally developed for B‐cell malignancies,^3^, ^4^ is now used in autoimmune diseases such as rheumatoid arthritis and multiple sclerosis. For pemphigus, rituximab has been highly effective in steroid‐sparing therapy for moderate to severe cases.^3^ In the study titled ‘First‐line Rituximab Combined with Short‐term Prednisone Versus Prednisone Alone for the Treatment of Pemphigus (Ritux3)’, originator rituximab, used as a first‐line treatment of pemphigus, showed a higher complete remission (CR) rate compared with the use of standard‐dose prednisone alone.^5^

Despite the favorable treatment effects of originator rituximab in patients with pemphigus, its high cost is a challenge. Therefore, rituximab biosimilars, such as Truxima, offer a potential solution by costing approximately 20% less than the originator in South Korea. However, it is important to note that biosimilars may not be identical to the originator. Studies examining the efficacy of biosimilars in the treatment of pemphigus has been conducted, but there are no studies comparing originator and biosimilar rituximab in the context of pemphigus.^6^ Thus, this study aims to compare the treatment effects of two rituximab agents, originator (MabThera, Roche ) and biosimilar (Truxima, Celltrion), in patients with pemphigus.

## 2 METHODS

### 2.1 Study population

From January 1, 2015, to December 31, 2021, we studied 321 patients with pemphigus receiving initial rituximab treatment at Gangnam Severance Hospital. To minimize the impact of protocol differences on the results, we included only patients treated with the rheumatoid arthritis protocol. A previous study has shown that a shorter time interval between disease onset and the first rituximab treatment leads to better clinical outcomes in terms of remission and total dose of steroid intake.^7^ Therefore, we further narrowed down the patient selection to those with a disease onset to first rituximab treatment duration of 1 year or less. The final study population comprised 52 patients in the MabThera group and 72 patients in the Truxima group, totaling 124 patients. CR was defined as maintaining a disease‐free state for at least 4 weeks with methylprednisolone at ≤2 mg/day. The CR attainment date was considered the first day of methylprednisolone administration at a dose of ≤2 mg/day.

### 2.2 Clinical parameters

We compared clinical baseline characteristics between the two groups, including sex, age, type of pemphigus, hospitalization rate, IVIG administration rate, and steroid dose at the date of rituximab treatment. A comparative analysis of enzyme‐linked immunosorbent assay titers for anti‐desmoglein 1 and anti‐desmoglein 3 was conducted between the two groups. The study included 44 patients in the MabThera‐treated group and 69 patients in the Truxima‐treated group. In addition, for comparison, Pemphigus Disease Area Index (PDAI) scores were calculated for patients. Data were available for 27 patients in the MabThera‐treated group and 55 patients in the Truxima‐treated group for this comparison. The analysis of B‐cell count before and after rituximab administration was conducted for 38 patients treated with Truxima. Complication analysis included events occurring within 90 days after rituximab administration. To compare the treatment effects of MabThera and Truxima, we set time to CR as the main outcome. Furthermore, we defined minor outcomes to compare efficacy and safety. These included the total steroid intake to CR, total steroid intake for 6 months following rituximab treatment, and infective complication rate potentially linked to rituximab use.

### 2.3 Statistical analysis

Statistical analysis was performed using SPSS version 25.0 software (IBM) and GraphPad (GraphPad Software). In the comparison process, paired *t*, Fisher, and chi‐square tests were employed. Data are expressed as means, percentages (numbers), or number‐to‐number ratios. A significance level of *p* < 0.05 was considered statistically significant.

### 2.4 Institutional review board approval status

The current study was reviewed and approved by the institutional review board of Gangnam Severance Hospital (approval #3‐2023‐0256).

## 3 RESULTS

Baseline characteristics were compared between the MabThera and Truxima groups (Table 1). General characteristics, including sex (50% vs 49% women; *p* = 0.879), type of pemphigus (PF:PV 10:42 vs 21:51; *p* = 0.207), and age at first diagnosis (*p* = 0.405) showed no significant differences. In addition, mean steroid dose at the first rituximab treatment (22.96 vs 21.75 mg/day; *p* = 0.583), hospitalization rate (44.2% vs 31.9%; *p* = 0.189), anti‐desmoglein 1 (174.23 vs 191.87; *p* = 0.412), anti‐desmoglein 3 (111.91 vs 158.63; *p* = 0.103), and PDAI score (31.37 vs 20.82; *p* = 0.051) did not differ. A statistically significant difference in IVIG administration rate (5.8% vs 27.8%; *p* = 0.002) was observed between the groups.

**TABLE 1:** Baseline characteristics of the study population.

	MabThera (*n* = 52)	Truxima (*n* = 72)	*p* Value
Sex (men:women)	26:26	35:37	0.879
Type of pemphigus (PF:PV)	10:42	21:51	0.207
Age at first diagnosis (year)			0.405
≤29	7 (13.5%)	5 (6.9%)	
30–39	7 (13.5%)	8 (11.1%)	
40–49	11 (21.2%)	16 (22.2%)	
50–59	12 (23.1%)	27 (37.5%)	
60+	15 (28.8%)	16 (22.2%)	
Methylprednisolone dose at first RTX (mg/day)	22.96	21.75	0.583
Hospitalization rate (%)	44.2	31.9	0.189
IVIG administration rate (%)	5.8	27.8	0.002

	MabThera (*n* = 44)	Truxima (*n* = 69)	*p* Value
Anti‐desmoglein 1 (IU/ml)	174.23	191.87	0.412
Anti‐desmoglein 3 (IU/ml)	111.91	158.63	0.103

	MabThera (*n* = 27)	Truxima (*n* = 55)	*p* Value
PDAI score	31.37	20.82	0.051

Abbreviations: IVIG, intravenous immunoglobulin; PDAI, Pemphigus Disease Area Index; PF, pemphigus foliaceus; PV, pemphigus vulgaris; RTX, rituximab.

**FIGURE 1:**
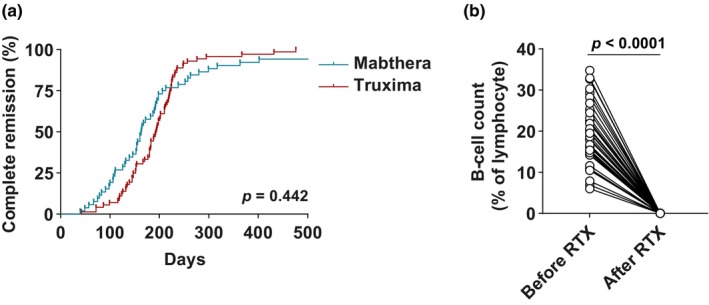
Time to complete remission after originator and biosimilar rituximab (RTX) and B‐cell count before and after administration of biosimilar RTX. (a) Kaplan–Meier curves show the time to complete remission in patients with pemphigus treated with MabThera (*n* = 52) and Truxima (*n* = 72). (b) The B‐cell count (percentage of lymphocyte) corresponds to data from 38 patients treated with Truxima.

Among the selected 124 patients, there was no significant difference in time to CR between the two groups (224.64 vs 192.92 days; *p* = 0.442) (Table 2, Figure 1a). Regarding the minor outcome, total steroid intake to CR did not show a significant difference (1927.5 vs 1666.06 mg; *p* = 0.361). In the case of mean total steroid intake for 6 months following rituximab treatment, a comparison was conducted excluding six patients (three from the MabThera group and three from the Truxima group) whose post‐rituximab treatment period of 6 months extended beyond the study duration. After the exclusion of these patients, no significant difference was observed (1289.22 vs 1351.22; *p* = 0.484). In 38 patients in the Truxima group with B‐cell data before and after rituximab treatment, a significant decrease in B‐cell count was observed after rituximab treatment (Figure 1b). The mean B‐cell count before and after treatment was 19.26% and 0.008%, respectively (*p* < 0.0001). Regarding complications, three patients experienced events. In the MabThera group, one patient developed sepsis 38 days after treatment and, in the Truxima group, two patients experienced pneumonia 57 days after treatment and herpes zoster infection 42 days after treatment, respectively.

**TABLE 2:** Comparison of outcome between the two groups.

	MabThera	Truxima	*p* Value
Time to CR (days)	224.63	192.92	0.442
Total methylprednisolone intake to CR (mg)	1927.5	1666.06	0.361
Total methylprednisolone intake for 6 months following RTX treatment (mg)	1289.22	1351.22	0.484

Abbreviations: CR, complete remission; RTX, rituximab.

## 4 DISCUSSION

The results of the present study demonstrate that biosimilar Truxima is not inferior to originator MabThera in the treatment of pemphigus. Regarding efficacy, there was no significant difference between the two groups when comparing time to CR. Indeed, CD19^+^ B lymphocyte depletions were observed after treatment of Truxima. Looking at other autoimmune diseases, in a study of patients with multiple sclerosis, comparing MabThera and Truxima, no significant differences were found in efficacy measures (lymphocyte count and magnetic resonance imaging activity) or safety profiles between the two medications.^8^ Comparing the two medications in patients with immune‐mediated thrombotic thrombocytopenic purpura, clinical parameters such as CD19 levels and ADAMTS13 activity did not differ significantly. Infusion reactions and infective complications were also comparable.^9^ Our results, along with those from studies on other autoimmune diseases, suggest Truxima as a cost‐effective alternative for patients with pemphigus indicated for MabThera. Cost utility analysis for PV treatment showed biosimilar rituximab as a first‐line option, reducing costs and increasing effectiveness compared with mycophenolate mofetil.^10^ These study findings strongly support the notion that Truxima could be an excellent option for the treatment of pemphigus.

In the comparison of baseline characteristics, all variables except IVIG administration rate showed no significant differences between groups. While IVIG has a beneficial effect in reducing disease severity in patients with pemphigus,^11^ there is no evidence that it shortens the time to CR after rituximab treatment.^12^ However, it should be considered that IVIG can remove pathogenic autoantibodies and provide normal circulating antibodies, which may affect total steroid intake and the occurrence of infection related to rituximab administration.

This study has some limitations. First, this is a retrospective, single‐center study. Second, data on B‐cell count were not available for all patients, limiting the investigation of B‐cell depletion to the Truxima group only. Third, the analysis of safety was limited. The study primarily focused on severe infections as complications, but the low frequency of such events made it challenging to perform statistical analyses.

In conclusion, the efficacy and safety of Truxima appear comparable to the originator, MabThera, in patients with pemphigus. Considering cost‐effectiveness, Truxima emerges as a favorable treatment option for the treatment of patients with pemphigus indicated as MabThera.

## CONFLICT OF INTEREST STATEMENT

None declared.
